# Genome and transcriptome based comparative analysis of *Tilletia indica* to decipher the causal genes for pathogenicity of Karnal bunt in wheat

**DOI:** 10.1186/s12870-024-04959-z

**Published:** 2024-07-16

**Authors:** Kalpana Singh, Pradeep Sharma, Sarika Jaiswal, Pallavi Mishra, Ranjeet Maurya, Senthilkumar K Muthusamy, MS Saharan, Rahul Singh Jasrotia, Jitender Kumar, Shefali Mishra, Sonia Sheoran, GP Singh, UB Angadi, Anil Rai, Ratan Tiwari, Mir Asif Iquebal, Dinesh Kumar

**Affiliations:** 1 Division of Agricultural Bioinformatics, ICAR-Indian Agricultural Statistics Research Institute, New Delhi, India; 2 Department of Bioinformatics, College of Animal Biotechnology, Guru Angad Dev Veterinary and Animal Sciences University, Ludhiana, India; 3 ICAR-Indian Institute of Wheat and Barley Research, Karnal, Haryana India; 4 ICAR-Indian Agricultural Research Institute, New Delhi, India; 5 ICAR-Central Tuber Crops Research Institute, Thiruvananthapuram, Kerala India

**Keywords:** Karnal bunt, Wheat, *Tilletia indica*, Whole genome assemblies, RNA-seq, Pathogenesis, Dikaryon

## Abstract

*Tilletia indica* Mitra causes Karnal bunt (KB) in wheat by pathogenic dikaryophase. The present study is the first to provide the draft genomes of the dikaryon (PSWKBGD-3) and its two monosporidial lines (PSWKBGH-1 and 2) using Illumina and PacBio reads, their annotation and the comparative analyses among the three genomes by extracting polymorphic SSR markers. The trancriptome from infected wheat grains of the susceptible wheat cultivar WL711 at 24* h*, 48*h*, and 7*d* after inoculation of PSWKBGH-1, 2 and PSWKBGD-3 were also isolated. Further, two transcriptome analyses were performed utilizing *T. indica* transcriptome to extract dikaryon genes responsible for pathogenesis, and wheat transcriptome to extract wheat genes affected by dikaryon involved in plant-pathogen interaction during progression of KB in wheat. A total of 54, 529, and 87 genes at *24hai*, 48*hai*, and 7*dai*, respectively were upregulated in dikaryon stage while 21, 35, and 134 genes of *T. indica* at *24hai*, 48*hai*, and 7*dai*, respectively, were activated only in dikaryon stage. While, a total of 23, 17, and 52 wheat genes at 24*hai*, 48*hai*, and 7*dai*, respectively were upregulated due to the presence of dikaryon stage only. The results obtained during this study have been compiled in a web resource called TiGeR (http://backlin.cabgrid.res.in/tiger/), which is the first genomic resource for *T. indica* cataloguing genes, genomic and polymorphic SSRs of the three *T. indica* lines, wheat and *T. indica* DEGs as well as wheat genes affected by *T. indica* dikaryon along with the pathogenecity related proteins of *T. indica* dikaryon during incidence of KB at different time points. The present study would be helpful to understand the role of dikaryon in plant-pathogen interaction during progression of KB, which would be helpful to manage KB in wheat, and to develop KB-resistant wheat varieties.

**Supplementary Information:**

The online version contains supplementary material available at 10.1186/s12870-024-04959-z.

## Introduction

Karnal bunt (KB) is a minor disease in wheat (*Triticum aestivum* [1], *T. durum*, *Secale cereal*, and *Triticosecale* [2, 3]), caused by a fungus *Tilletia indica* [4]. Although, it does not significantly impact yield loss (0.3-0.5%), the reduction in grain quality causes far more economic loss due to the significant effect on grain marketability in wheat producing countries across the globe [5, 6]. *T. indica*, is a heterothallic fungus with bipolar incompatibility controlled by multiple alleles at one locus [7–10], which form a pathogenic dikaryophase. *T. indica* survives as diploid teliospores, germinating and producing haploid primary allantoid sporidia (basidiospores). Two compatible strains of allantoid sporidia conjugate immediately on the promycelium, and produce dikaryotic infectious hyphae or secondary sporidia that systemically infect wheat seedlings [7, 11–13]. In the present study, the main focus was on dikaryotic infectious hyphae.

Till today, various studies related to *T. indica* involving genome assembly, genomic variability, and genes associated with pathogenesis have been published. A few draft genomes of *T. indica* have been published by now, which are of PSWKBGH-1 and PSWKBGH-2 monosporidial lines [13], highly virulent Karnal, TiK isolate [14], and RAKB_UP_1 isolate [15]utilized hyphae germinated from teliospores. Gurjar et al., 2021 [16] also performed a study for genetic variability of *T. indica*, and in another study, Gurjar et al., 2022a [17] performed an analysis of identification of SSR markers utilizing the same biological samples as Gurjar et al., 2019 [15]. Mishra et al., 2019 [18] provided a comparative analysis of genomes and pathogenesis related genes of monosporidial lines and hyphae germinated from teliospore. Similarly, only a few transcriptome data analyses of *T. indica* have reported so far. Gurjar et al., 2018a [11] studied regulation of genes from *T. indica* grown from germination of teliospores. Most recently, a study by Gurjar et al., 2022b [19] extracted differentially expressed genes of RAKB_UP_1 isolate of *T. indica* utilizing draft genome of a non-pathogenic monosporidial line for mapping.

It is evident that all previous studies related to *T. indica* were based on either monosporidial lines or hyphae grown from germination of teliospores. Therefore, in the present study, true pathogenic dikaryon hyphae, termed as PSWKGD-3 and its monosporidial lines PSWKBGH-1 and 2 were identified and isolated from KB infected wheat grains of susceptible cultivar, WL711 to grow in pure culture. Further, this culture of PSWKGD-3 was utilized as biological sample to analyse dikaryon stage of *T. indica*. First, whole genome assembly of PSWKBGD-3 was performed along with PSWKBGH-1 and 2, which were announced in 2016 [13]. Further, genome-wide SSR mining was performed along with extraction of polymorphic SSRs among three genomes (first study); which could be used in identification of isolates and to determine their genetic variability. Additionally, the present study utilized hyphae of PSWKBGD-3 and PSWKBGH-1, 2 to inoculate wheat seeds (WL711) to isolated RNA at 24*hai*, 48*hai* and 7*dai* to perform transcriptome analyses for both wheat and *T. indica* transcripts to understand host-pathogen interaction during progression of KB. This is the first study on dikaryon stage of *T. indica* at the level of genome and transcriptome to determine *T. indica* genes causing pathogenesis and wheat genes affected during progression of KB in wheat. The results from the study were compiled in a first web resource for *T. indica*, called TiGeR (http://backlin.cabgrid.res.in/tiger/), which could be used by the research community. The present study would be helpful to understand the pathogenic dikaryon stage and its role in plant-pathogen interaction during progression of KB, which would be helpful to manage KB in wheat, and to develop KB-resistant wheat varieties.

## Materials and methods

### *T. indica* monosporidial lines and their dikaryon cultures

*T. indica* was isolated from naturally infected kernels of susceptible variety of wheat from Karnal to get monosporidial lines (i.e., PSWKBGH-1 and 2). The basidiospores from the germinating teliospores were teased on separate agar plates with drops of sterile water. The water agar block with single allantoid, i.e., germinating spore was separated aseptically from the plate and transferred to a new petri plate with PDA media. These petri plates, containing single spore, were sealed with parafilm followed by incubation in a BOD incubator (18 ± 2 °C) for 20 days under alternate light and dark conditions. After the formation of colonies, the Ms lines were multiplied on PDA slants and assigned numbers as PSWKBGH-1 and PSWKBGH-2. For dikaryon stage culture (PSWKBGD-3), allantoid sporidia of both monosporidial lines were germinated from separate cultures on the same agar medium [12, 13, 20]. Supplementary Fig. 1 indicates confocal microscopy image showing fungal mycelium growth and spore in developing wheat grain. For electron microscopic analysis, the fungal mycelium from individual purified single-spore cultures were placed under a fixative consisting of 2.5% glutaraldehyde and 2% paraformaldehyde prepared in 0.1 M sodium phosphate buffer pH 7.2. The fixed sample was analyzed by Transmission Electron Microscope (TEM) (JEOL2100F) at Advanced Instrumentation Research Facility (AIRF) at Jawaharlal Nehru University (JNU), New Delhi. First, calcofluor white dye was used to stain the control and KB infected wheat sample to identify the apparent infection. To visualize the early events of fungal infection, confocal laser scanning microscopy (CLSM) was performed at IISER, Mohali. Fungus staining was performed using 5% blue cotton in lactophenol for 20 min. Slides were prepared separately for treated and control samples and viewed under an upright confocal microscope. Confocal image stacks of 30–40 sections spaced ∼ 1.5 m with a scan speed of 400 milliseconds, 512 × 512 pixels/image were collected using a 40X objective (Leica SP8, Germany). The 488 nm laser line coupled with an adjustable bandwidth filter of 500–540 nm was applied to acquire images.

### In vivo pathogenicity test of monosporidial lines and their dikaryon stage

Monosporidial lines (PSWKBGH-1 and 2) and dikaryon (PSWKBGD-3) were subjected to pathogenicity test at the ICAR-Indian Institute of Wheat and Barley Research (IIWBR) experimental farm in Karnal, India, to be confirmed in wheat varieties susceptible to KB (WL711 and WH542). Fifteen ear heads were inoculated at the boot stage by the syringe inoculation method [21] with dikaryon and both monosporidial lines grown in polyhouse conditions, where, proper temperature and relative humidity were maintained for proper disease development. Monokaryon hyphae fuse by anastomosis, creating primary and secondary sporidia. Primary sporidia are filiform-like threads that do not cause infection. Secondary sporidia are formed from primary sporidia, which are called allantoid or banana-shaped spores, produce dikaryon. However, in the dikaryon stage, PSWKBGD-3 (produced from PSWKBGH-1 and 2) cause disease. Disease data recorded as % KB incidence (I) and coefficient of infection (CI) were calculated based on various degrees of disease (Table 1).

**Table 1:** Pathogenicity test of monosporidial lines and dikaryon stages of *T. indica* tested during 2014-15 and 2015-16 on artificially inoculated susceptible wheat cultivars WL711 and WH542

T. indica lines	2014-15				2015-16			
	WH542		WL711		WH542		WL711	
	% I	CI	% I	CI	% I	CI	% I	CI
PSWKBGH-1 (Monosporidial)	0	0	0	0	0	0	0	0
PSWKBGH-2 (Monosporidial)	0	0	0	0	0	0	0	0
PSWKBGD-3 (Dikaryon PSWKBGH-1 × 2)	14.67	4.12	7.40	2.77	12.62	4.29	4.82	1.20

%I = Percent Incidence and CI = Coefficient of Infection of Karnal bunt

### Sample collection, DNA isolation, and whole genome sequencing

The vegetative mycelia of PSWKBGH-1, 2 and PSWKBGD-3 were inoculated in potato dextrose broth (Difco, USA) in BOD for 20 days. Mycelial beads were harvested from each of PSWKBGH-1 and 2 lines, and one sample from PSWKBGD-3. Total DNA from samples using a DNeasy Plant Maxi kit (Qiagen India Pvt. Ltd., India) according to the manufacturer’s instructions. The quality of the isolated DNA was checked using a NanoDrop D-1000 spectrophotometer (NanoDrop Technologies, Inc., USA) and a qubit fluorometer. Further, three libraries of Illumina NextSeq500 (150 × 2 Pair-end reads) (Illumina Inc. USA) were obtained for all the three lines; while, two libraries of PacBio single molecule real time (SMRT) sequencing (RSII) platform (Pacific Biosciences of California, Inc. USA) using library construction strategy CLR were also obtained for PSWKBGH-1 and 2.

### Genome assembly, assessment, annotation and comparative analysis

**Fig. 1:**
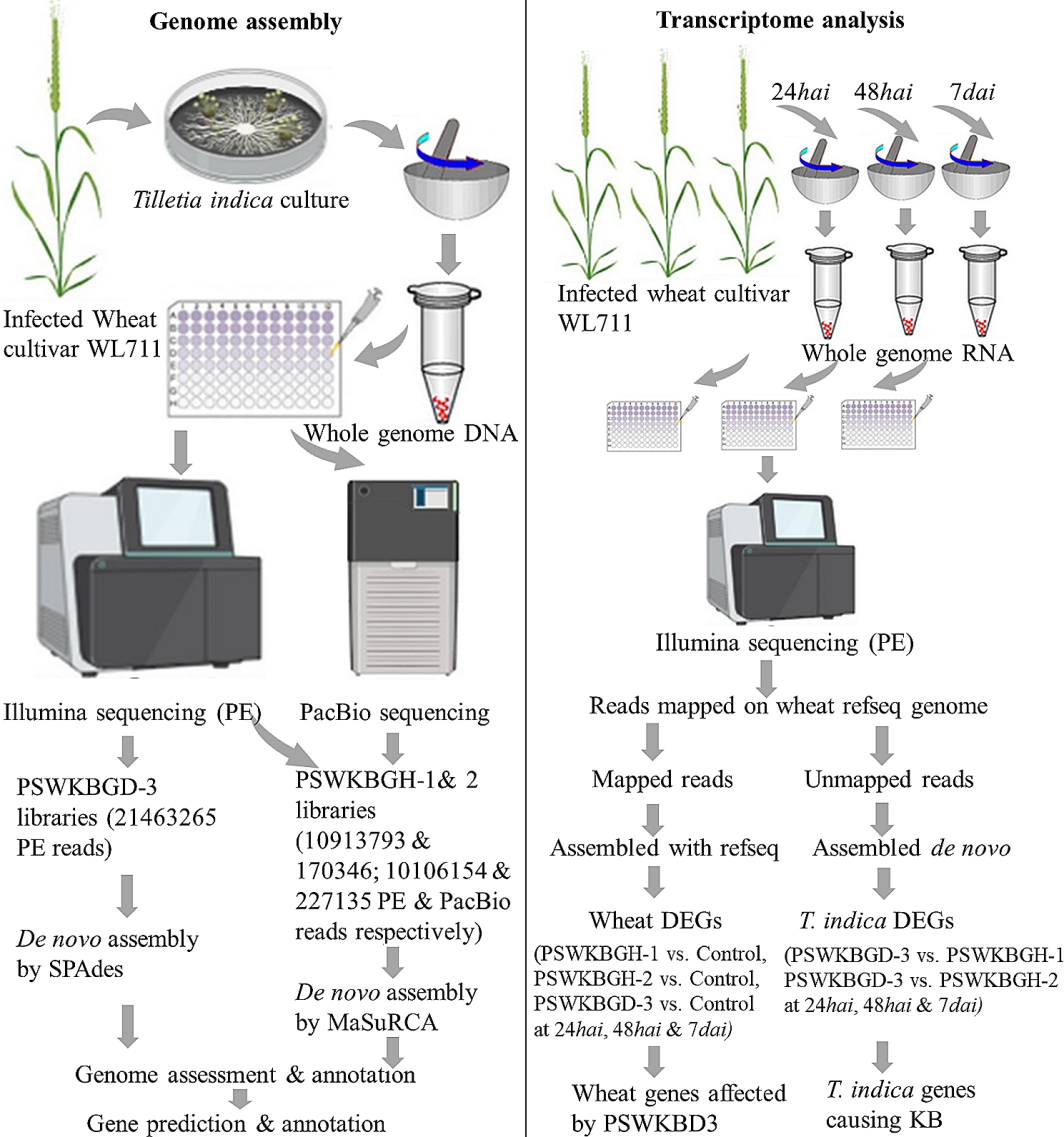
Schematic diagram of sequencing, whole genome assembly and transcriptome analysis for *T. indica* dikaryon stage and its monosporidial lines

The standard protocol for quality check of raw reads for the three libraries were followed using FastQC-toolkit (v2.2) [22]. The raw reads were trimmed for adapters along with low-quality bases towards 3’end using our in-house PERL script. The reads with lengths < 20 bp were also discarded. *De novo* assembly was performed for PSWKBGH-1 and 2 using MaSuRCA [23] as both, Illumina and PacBio reads were available. Since only Illumina PE reads were available for PSWKBGD-3, *de novo* assembly was carried out using the SPAdes [24] and assembled contigs were scaffolded using the SSPACE [25] (Fig. 1). The detailed assembly statistics was computed using QUAST [26]. The quality assessment was done using BUSCO [27], 1764 BUSCO groups of Basidiomycota from basidipmycota_odb10 for all three assemblies were obtained. The genes were predicted for all assemblies using AUGUSTUS [28] and annotation of predicted genes was performed using BLAST [29] against the nearest reference organisms. These genome assemblies were submitted to NCBI under bioproject PRJNA325874, i.e., PSWKBGH-1 (GCA_001689995.1), PSWKBGH-2 (GCA_001689945.1) and PSWKBGD-3 (GCA_001689965.1 (https://www.ncbi.nlm.nih.gov/bioproject/PRJNA325874). Further, SSRs were mined from all assembled genomes using MISA [30] and compared to find monomorphic and polymorphic SSRs along with unique SSRs for each assembly. The 5’ and 3’ 20 nucleotide flanking sequences of each SSR were matched between SSRs of two assemblies to find monomorphic and polymorphic SSRs using PERL scripts.

### Sample collection, RNA isolation and library preparation

The plant samples of susceptible wheat cultivar (WL711), inoculated with monosporidial *T. indica* lines (PSWKBGH-1 and PSWKBGH-2) and their dikaryon (PSWKBGD-3) in two replicates along with the control treated with distilled water were collected at 24*hai*, 48*hai* and 7*dai* and stored at -80 °C (Fig. 1). Total RNA was isolated from each sample using TRIzol™ Reagent (ThermoFisher Scientific Catalog number: 15,596,026) according to the manufacturer’s instructions. It was treated with RNase-free DNase I (Invitrogen solutions, USA) for 30 min at 37 °C to remove residual DNA. RNA quantity and quality were determined using the Agilent Technologies 2100 Bioanalyzer (Agilent Technologies Inc., USA). RNA samples with RNA integrity number (RIN) value 7.5 were used for sequencing. Beads with oligo (dT) were used to isolate poly(A) mRNA from total RNA. A total of 21 RNA-seq libraries at 24*hai*, 48*hai*, and 7*dai* were constructed from these poly(A) mRNA using the TruSeq RNA Sample Preparation Kit (Illumina Inc., USA) using 3 µg of total RNA. Constructed libraries were sequenced on Illumina HiSeq 2500 platform and 2 × 100 bp PE reads were generated.

### Data pre-processing and transcriptome analysis

For pre-processing of raw reads, Cutadapt v1.8.1 [31] and Sickle v1.3 [32] were used, where reads with ambiguous bases ‘N’ and phred score < 30 were removed. Silva database [33] was used for rRNA sequencing data. The cleaned reads were mapped and assembled with IWGSC RefSeq2.1 of *Triticum aestivum* cv. Chinese Spring (https://urgi.versailles.inra.fr/download/iwgsc/IWGSC_RefSeq_Assemblies/v2.1/) using Bowtie2 v. 2.2.9 [34] and StringTie2 [35]. Mapped transcripts were used to study the wheat genes affected by the infection caused by monosporidial lines and dikaryon of *T. indica*. The unmapped reads were assembled *de novo* using *Trinity* assembler [36] followed by CD-HIT-EST tool for removal of redundant transcripts [37]. Obtained transcripts unmapped with wheat RefSeq 2.1 were supposed to be transcripts of *T. indica* and used to study the *T. indica* genes involved in causing pathogenicity in wheat. Further, read count analysis was performed by using RSEM [38] and differential expression analysis was performed by DEseq2 [39] to extract differentially expressed regions (DERs) with log2FC (foldchange) ≥ 2 and p-value < 0.05 in both, wheat and *T. indica* transcripts. For the annotation of wheat DERs, annotation file of high confidence genes of IWGSC RefSeq2.1 (https://urgi.versailles.inrae.fr/download/iwgsc/IWGSC_RefSeq_Annotations/v2.1/) was used to extract differentially expressed genes (DEGs) of wheat, while in case of *T. indica* DERs, Blastx was performed against non-redundant protein (NR) database with e-value 10^− 5^ and identity cut-off of 40% to obtain DEGs of *T. indica*. Further, *T. indica* DEGs were annotated and analysed for the secretory and effector genes as pathogenicity related genes using DeepLoc v1.0, SignalP v6.0, TargetP v2.0, TMHMM v2.0, and EffectorP-fungi v3.0 [40–44]. Finally, predicted effectors and signal peptides were mapped by BLASTp [29] with PHI-base (pathogen-host interactions) database [45] to further, characterized the effectors as hypervirulence, plant avirulence determinant and lethal.

### Development of *Tilletia indica* genomic resource (*TiGeR*)

The results of this study were catalogued in the form of relational database, *T. indica* genomic resource (*TiGeR)* based on three-tier architecture, comprising of client, middle and database tiers. The database was developed in MySQL (https://www.mysql.com/), its web-interface was prepared in PHP (https://www.php.net/) and HTML, and finally, hosted on Apache2 server (https://httpd.apache.org/). Data retrieval can be done by first generating query from user’s system to webserver, which is sent to MySQL database. The database response is generated and sent to web-interface and finally, web-server response is sent to user. *TiGeR* includes two broader resources, *i.e., genomic resource*, that includes all the genomic information extracted in the study (*namely*, genes and SSRs from all assemblies) and the *transcriptome resource* that includes differentially expressed wheat and *T. indica* genes during progression of KB in wheat.

## Results

### Genome assembly, assessment, annotation and comparative analysis

The details of raw reads of WGS libraries from Illumina and PacBio sequencing platforms are provided in Supplementary Table 1. *De novo* genome assembly was performed on WGS libraries of monosporidial lines of *T. indica* and their dikaryon. Statistics of genome assemblies showed N50 as 200,513, 132,740 and 13,494 for PSWKBGH-1, 2 and PSWKBGD-3, respectively. A total of 367, 482 and 8812 scaffolds were generated along with 78.3%, 76.2% and 90.3% BUSCO (complete) along with 14.4%, 16.4% and 6.5% BUSCO genes were missing in PSWKBGH-1, 2, and PSWKBGD-3, respectively, which may be due to insufficient quality and depth of the sequencing data leading to the missing orthologs or incomplete transcripts/ gene models. Genome sizes of 37,460,344, 37,216,861 and 43,810,954 bp were observed for PSWKBGH-1, 2, and PSWKBGD-3, each with ∼ 54% CG content (Table 2).

**Table 2:** Detailed statistics of genome assemblies of monosporidial lines (PSWKBGH-1, 2) and dikaryon stage (PSWKBGD-3) along with BUSCO quality assessment, predicted genes, proteins and SSRs

General features	PSWKBGH-1	PSWKBGH-2	PSWKBGD-3
Contigs generated	367	482	8812
Total GC Content	54.58%	54.59%	54.56%
Maximum contig length	924,144	618,870	112,423
Minimum contig length	2276	1269	500
Average contig length	102,280	77,499	4971
Median contig length	53,804	47,324	3252
Total contig length	37,460,344	37,216,861	43,810,954
Total non-ATGC characters	57	25	49,017
Contigs > = 200 bp	367	482	8812
Contigs > = 1 Kbp	367	482	6201
Contigs > = 10 Kbp	357	446	1151
N50	200,513	132,740	13,494
BUSCO (Complete)	78.3–82%	76.2%	90.3%
BUSCO (Complete-single)	77.7–81.2%	74.8%	81.3%
BUSCO (Complete-duplicate)	0.6–0.8%	1.4%	9.0%
BUSCO (Fragmented)	7.3–10.6%	7.4%	3.2%
BUSCO (Missing)	14.4–7.4%	16.4%	6.5%
Predicted genes	10,203	10,304	13,413
Annotated genes	9718	9831	12,768
Encoded unique proteins	7556	7570	9048
Annotated with *Tilletia* species	9677	9781	12,696
Annotated with other than *Tilletia*	41	50	72
SSRs	6347	6164	6938

**Fig. 2:**
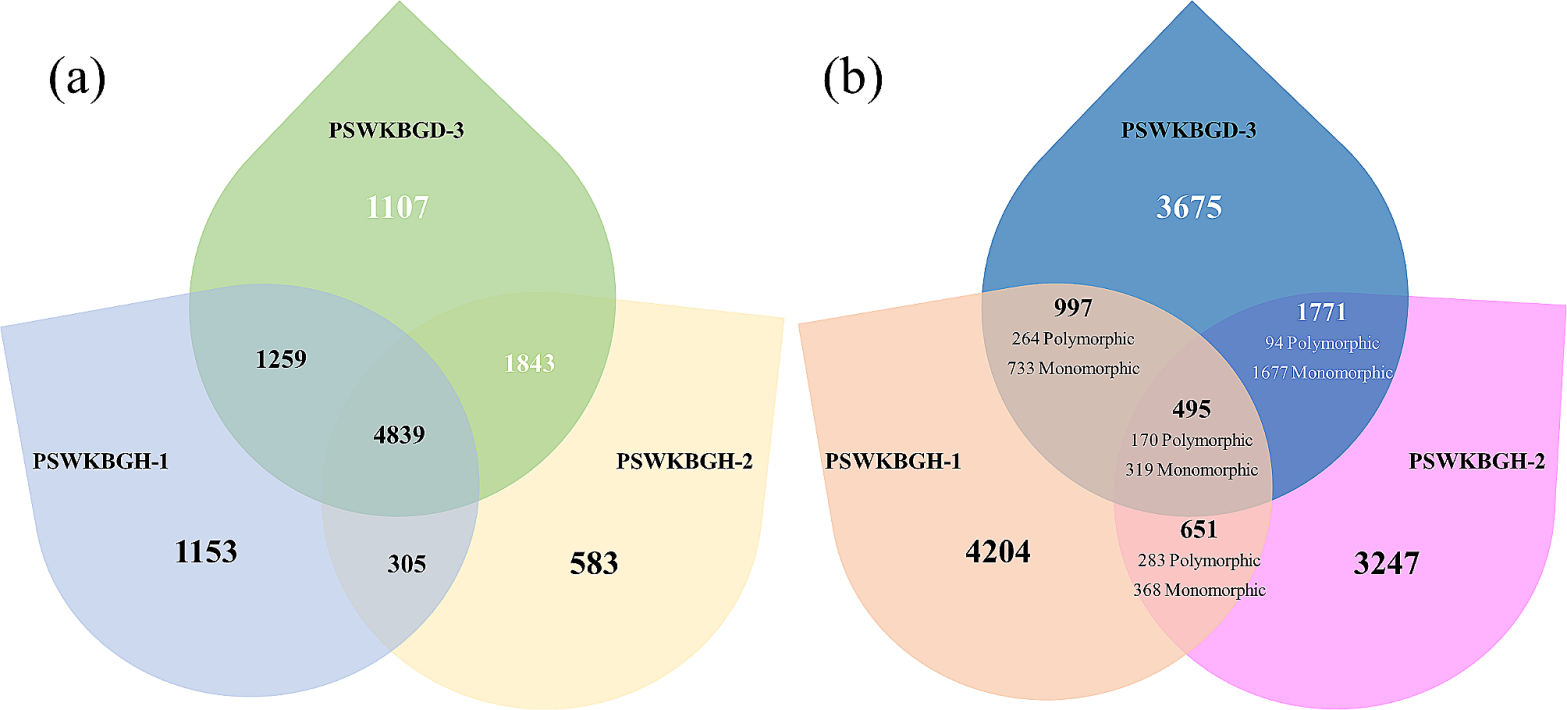
(**a**) Unique and shared protein of annotated genes; (**b**) Unique and shared SSRs (monomorphic and polymorphic) among monosporidial lines (PSWKBGH-1, 2) and their dikaryon stage (PSWKBGD-3) assemblies

**Fig. 3:**
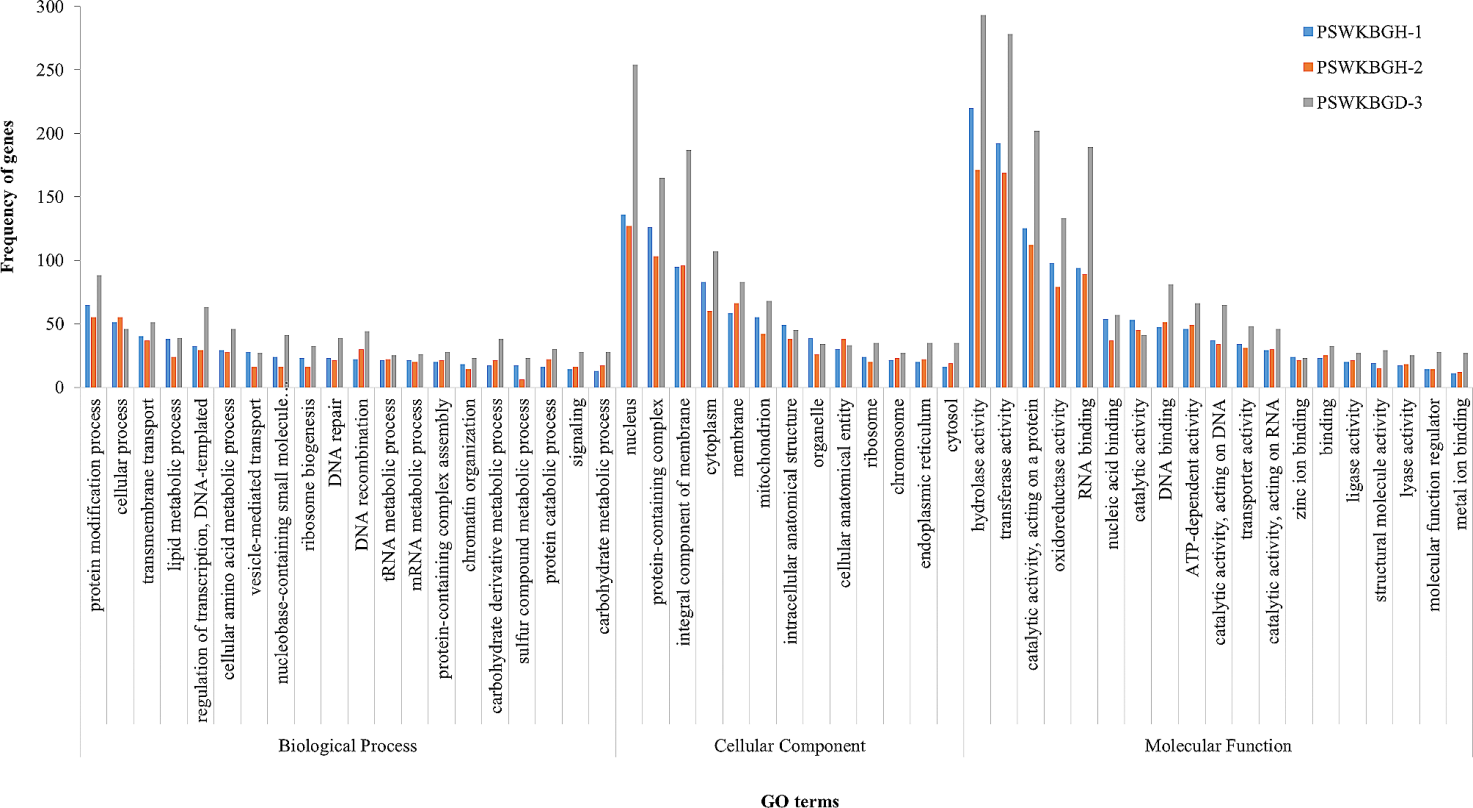
Bar chart representing GO terms associated with unique > 15 genes annotated in PSWKBGH-1, PSWKBGH-2 and PSWKBGD-3 genomes

A total of 10,203, 10,304 and 13,413 genes were extracted, out of which 9718, 9831 and 12,768 annotated proteins were found with 7556, 7570, and 9048 functions in PSWKBGH-1, 2 and PSWKBGD-3, respectively. It was observed that dikaryon PSWKBGD-3 has more genes in comparison to its monosporidial lines (Table 2). The Venn diagram in Fig. 2 (a) reveals 4839 common proteins among the three assemblies, while 1153, 583 and 1107 proteins were unique to PSWKBGH-1, 2 and PSWKBGD-3, respectively. The GO terms of the unique genes annotated in PSWKBGH-1, 2 and PSWKBGD-3 genomes are shown in Fig. 3. Under the category of cellular components, the nucleus was the most common, followed by proteinaceous complexes and integral membrane components in all three genomes. The common GO terms in the biological process category were protein modification process, cellular process, and transmembrane transport in all three genomes.

**Fig. 4:**
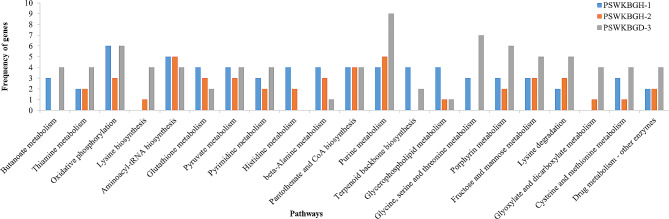
KEGG pathways associated with > 3 genes annotated in PSWKBGH-1, 2 and PSWKBGD-3 genomes

KEGG pathway[47] analysis reveals phenylalanine, tyrosine, and tryptophan biosynthesis as the most abundant pathway, followed by amino sugar and nucleotide sugar metabolism and oxidative phosphorylation activated by genes annotated in PSWKBGH-1. In the case of PSWKBGH-2, the abundant pathways were amino sugar and nucleotide sugar metabolism; aminoacyl-tRNA A biosynthesis; and purine metabolism activated by annotated genes. The main metabolic pathways were purine metabolism; Amino sugar and nucleotide sugar metabolism; and glycine, serine and threonine metabolism activated by genes annotated in PSWKBGD-3 (Fig. 4).

A total of 6347, 6164, and 6938 SSRs (Table 3) were mined from PSWKBGH-1, 2, and PSWKBGD-3 assemblies, respectively, of which trinucleotide SSR repeats were the most abundant, followed by mono- and dinucleotide SSR repeats (Supplementary Table 2). Also, 3709, 2752, and 3180 SSRs were unique to PSWKBGH-1, 2, and PSWKBGD-3 assemblies, respectively. A total of 170 SSRs were found to be polymorphic and 319 SSRs monomorphic among all three assemblies (Fig. 2b).

**Table 3:** Important wheat genes upregulated and downregulated due to PSWKBGD-3 only during progression of Karnal bunt by *T. indica* in wheat at different transitions of time. The numbers in brackets are the number of differentially expressed genes.

Expression	Transitions	Function of wheat DEGs affected by PSWKBGD-3
Upregulated due to PSWKBGD-3	24*hai* (19)	PIF1 helicase; Ribosomal protein S12; Wall-associated receptor kinase 2; Oligomeric Golgi complex subunit 2; Protein HOTHEAD; ATP synthase subunit C; Homeobox-leucine zipper protein HOX15; Protein argonaute 4B; Peptidyl-prolyl cis-trans isomerase; Pentatricopeptide repeat-containing protein; Hypothetical protein; Acetyltransferase; protein Rf1
	48*hai* (15)	ATP synthase subunit 9; cysteine-rich receptor-like protein kinase; Two disease resistance protein RGA1; 2’-deoxymugineic-acid 2’-dioxygenase; Xyloglucan endotransglycosylase/ hydrolase protein 8; Glycosyltransferase; Disease resistance protein RPM1; Disease resistance protein RGA2; Hypothetical protein; Protein UPSTREAM OF FLC
	7*dai* (46)	Protein CHUP1, chloroplastic; CASP-like protein 5C1; CLP protease regulatory subunit; fatty acid desaturase DES2-like isoform X2; Nucleolar and coiled-body phosphoprotein 1; Histidine-containing phosphotransfer protein 3; Non-specific lipid-transfer protein; Nine predicted protein; AAA-ATPase; Uncharacterized aarF domain-containing protein kinase; Protein COFACTOR ASSEMBLY OF COMPLEX C SUBUNIT B; Mediator of RNA polymerase II transcription subunit 19b; Polycomb group protein; Two coatomer subunit beta; Maspardin; Two STAY-GREEN LIKE; Transcription factor SPATULA; Exopolygalacturonase; Uncharacterized protein; Protein HGV2; Bifunctional purine biosynthesis protein purH; Two hypothetical proteins; Zinc finger protein VAR3; Three disease resistance RPP13 protein; AdoMet-dependent rRNA methyltransferase spb1; Transmembrane protein 53; Caffeine synthase 1; PS I reaction center subunit VI; Chlorophyll a-b binding protein 1; Protein NETWORKED 2 A; Zinc finger protein 7; Cytochrome b5; UPF0481 protein
	24*hai*, 48*hai*(01)	Disease resistance protein
	48*hai*,7*dai*(01)	1,4-dihydroxy-2-naphthoyl-CoA thioesterase 1
	24*hai*, 7*dai*(03)	tRNA pseudouridine synthase A; Pleiotropic drug resistance protein 12; 6-phosphofructokinase 2
Down-regulated due to PSWKBGD-3	24*hai* (22)	Two wall-associated receptor kinase 6; Disease resistance protein RGA3; protein DETOXIFICATION 16; Disease resistance RPP13 protein; LRR receptor-like serine/threonine-protein kinase; ABC transporter F family member 4; NBS-LRR protein; SnTox1 sensitivity protein; Polyol transporter 5; E3 ubiquitin-protein ligase; NAC domain-containing protein; U3 small nucleolar RNA-associated protein; 7-Deoxyloganetin glucosyltransferase; Four predicted proteins; Metallothionein protein; Cytochrome c oxidase assembly protein; Glutathione S-transferase
	48*hai* (19)	Protein accelerated cell death 6; vegetative cell wall protein gp1; Peptidyl-prolyl cis-trans isomerase; RNA-binding protein; Serine/arginine-rich splicing factor; Disease resistance protein RGA2; Glycine-rich cell wall structural protein; CBL-interacting protein kinase 26; cinnamoyl-CoA reductase 1; Wall-associated receptor kinase 5; Callose synthase 6; Pre-mRNA-processing-splicing factor 8; Ribosomal protein S12; Short-chain dehydrogenase/reductase 2b; NADH-plastoquinone oxidoreductase subunit 2; CCA tRNA nucleotidyltransferase 2; GPI mannosyltransferase 3; Two Predicted proteins
	24*hai*, 48*hai*(01)	High mobility group B protein 4
	24*hai*,7*dai*(05)	R3H domain-containing protein 2; BTB/POZ domain-containing protein; Ankyrin repeat, PH and Sect. 7 domain containing protein; VQ motif-containing protein 22
	24*hai*, 48*hai*, 7*dai* (01)	Nudix hydrolase 9

### Transcriptome analysis

A sum of 21 RNA libraries were prepared from the samples collected from the susceptible wheat cultivar, WL711 inoculated with monosporidial *T. indica* lines and their dikaryon (in two replicates) along with controls (in single replicate) at 24*hai*, 48*hai* and 7*dai*. The detailed statistics of raw reads (average ∼ 97,179,468), cleaned reads (average ∼ 2,896,709), GC% (average ∼ 47) of cleaned reads and transcripts (average ∼ 85,477) are given in Supplementary Table 3.

### Differentially expressed wheat genes affected by ***T. indica*** infection

**Fig. 5:**
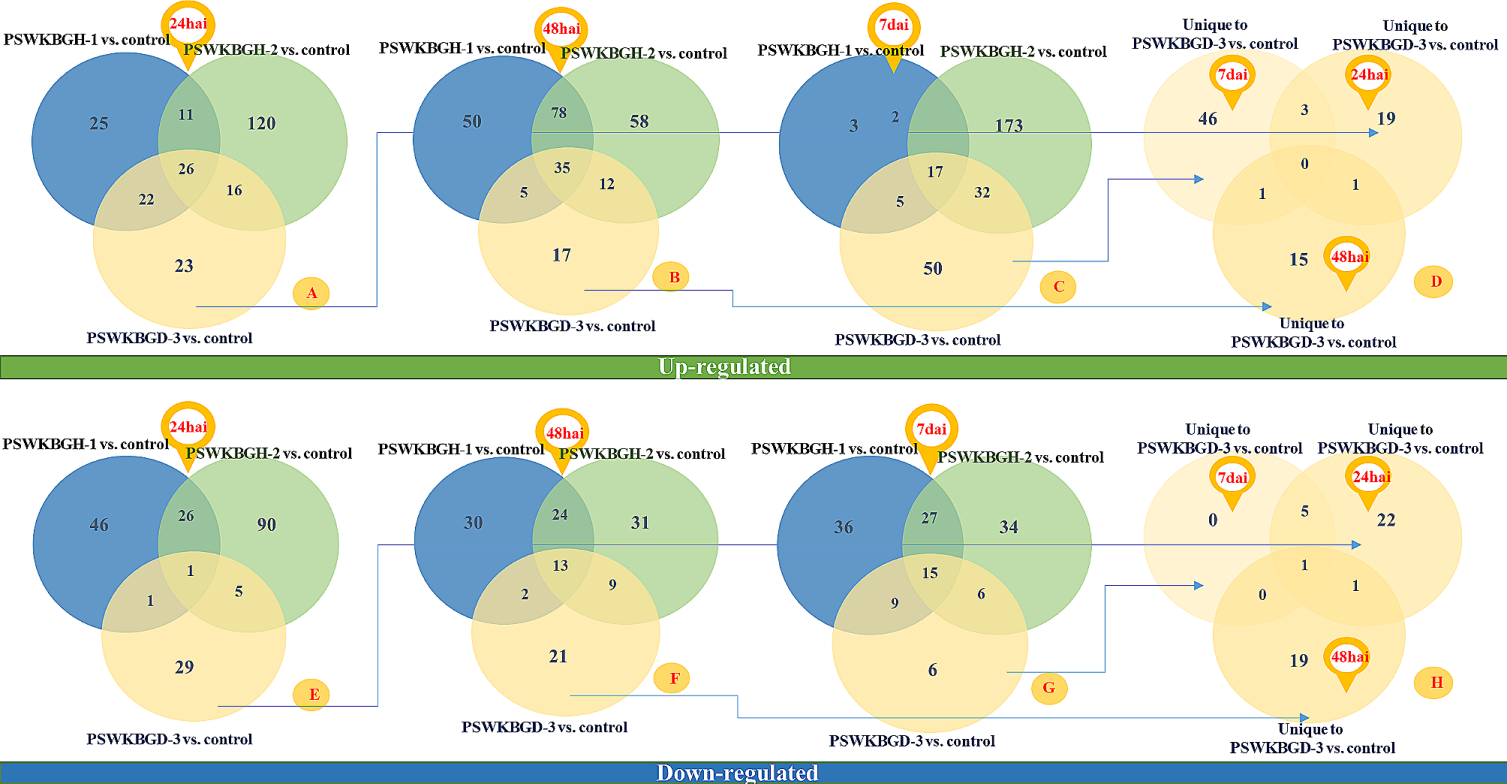
Common upregulated wheat DEGs extracted from affected susceptible wheat cultivar WL711 inoculated with monosporidial lines (PSWKBGH-1 and 2) and dikaryon stage (PSWKBGD-3) of *T. indica* in comparison to control at (**A**) 24*hai*, (**B**) 48*hai* and (**C**) 7*dai* and downregulated DEGs at (**E**) 24*hai*, (**F**) 48*hai* and (**G**) 7*dai*. (**D**) Unique upregulated DEGs in PSWKBGD-3 vs. control treatment pair at 24*hai*, 48*hai* and 7*dai* (**H**) Unique downregulated DEGs in PSWKBGD-3 vs. control treatment pair at 24*hai*, 48*hai* and 7*dai*

Transcripts mapped with wheat RefSeq v2.1 were used to find differentially expressed wheat genes of affected susceptible wheat cultivar WL711 inoculated with monosporidial lines (PSWKBGH-1 and 2) and dikaryon stage (PSWKBGD-3) of *T. indica*. Differentially expressed regions and genes were extracted from nine combinations as shown in Supplementary Tables 4 and it was found that most DEGs were extracted from PSWKBGH-2 in comparison to control at 7*dai* and the least from PSWKBGH-1 in compared to control at 7*dai* extracted from the annotation of DERs. It was also observed that in PSWKBGH-1, the maximum number of up-regulated DEGs were found at 48*hai*, while in PSWKBGD-3 and PSWKBGH-2 at 7*dai*. However, in PSWKBGH-1, maximum number of down-regulated DEGs were found at 7*dai*, in PSWKBGH-2 and PSWKBGD-3 at 24*hai*. Figure 5A, B, C shows more expression of up-regulated DEGs in PSWKBGH-1, 2 and PSWKBGD-3 in comparison to control at 24*hai*, 48*hai* and 7*dai* respectively, at certain time only (shown by unique DEGs), however, few regulated the infection over time causing transition from one stage to other (shown by overlapped DEGs) during progression of pathogenesis. Similarly, Fig. 5E, F, G shows the same for downregulated DEGs. Figure 5D for upregulated DEGs (unique DEGs from Fig. 5A, B and C) and Fig. 5H for downregulated DEGs (unique DEGs from Fig. 5E, F and G) show that the most of DEGs were expressed at a certain time extracted from unique DEGs in PSWKBGD-3 vs. Control in comparison to PSWKBGH-1 and 2 vs. Control at 24*hai*, 48*hai* and 7*dai.*

Functional annotation was performed on 48 downregulated (Fig. 5D) and 85 up-regulated (Fig. 5H) wheat DEGs only expressed in PSWKBGD-3 vs. control in comparison to PSWKBGH-1 and2 vs. control at 24*hai* (Table 3). Table 3 lists the functions of the DEGs expressed during transition from 24*hai* to 7*dai*, 24*hai* to 48*hai* and 48*hai* to 7*dai*, along with unique DEGs expressed at given time after the inoculation of PSWKBGD-3. A separate KEGG pathway analysis suggested that One carbon pool by folate, Purine metabolism, and Pentose and glucuronate interconversions pathways were affected the most by upregulated DEGs; while, Ubiquinone and other terpenoid-quinone biosynthesis, and Oxidative phosphorylation pathways were affected the most by downregulated DEGs in wheat.

### Differentially expressed *T. indica* genes causing infection in wheat

**Fig. 6:**
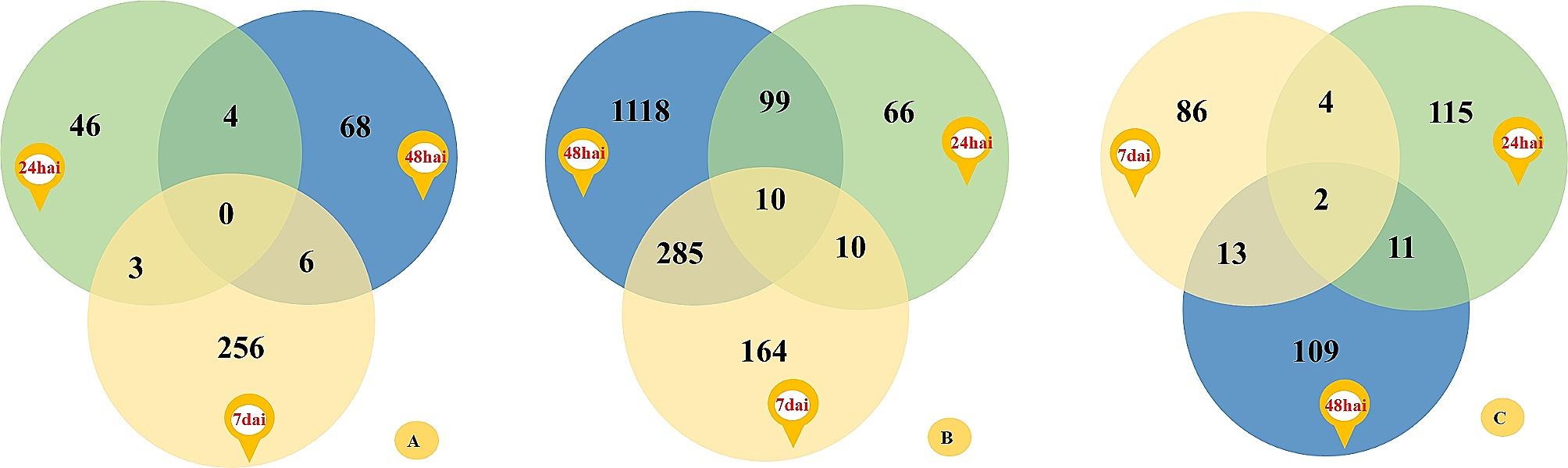
*T. indica* DEGs (**a**) expressed only; (**b**) upregulated; (**c**) downregulated in PSWKBGD-3 dikaryon of *T. indica* in comparison to both PSWKBGH-1 and 2 monosporidial lines at 24*hai*, 48*hai* and 7*dai* (extracted from Supplementary Fig. 4)

Transcripts not mapped with wheat RefSeq v2.1 were *T. indica* transcripts and were used to find differentially expressed *T. indica* genes, which are involved in the growth of monosporidial lines and dikaryon of *T. indica* in wheat. Differentially expressed regions and genes were extracted from 06 treatment pairs, as shown in Supplementary Figs. 2 and 3, respectively. *T. indica* DEGs expressed only in PSWKBGD-3, up- and down-regulated DEGs in PSWKBGD-3 in comparison to PSWKBGH-1 and 2 (from PSWKBGD-3 vs. PSWKBGH-1 and PSWKBGD-3 vs. PSWKBGH-2) at 24*hai*, 48*hai* and 7*dai* are shown in Supplementary Fig. 3. The most DEGs were extracted from PSWKBGD-3 in comparison to PSWKBGH-1 at 48*hai* and the least were extracted from PSWKBGD-3 in comparison to PSWKBGH-1 at 24*hai* (Supplementary Fig. 3). A greater number of *T. indica* genes were up-regulated in PSWKBGD-3 in comparison to PSWKBGH-1 and 2 monosporidial lines from 24*hai* to 7*dai* (Supplementary Fig. 3). The frequency of *T. indica* DEGs, which are only expressed in PSWKBGD-3, increased approximately with the time of inoculation. Common and unique DEGs that are up-regulated, down-regulated and expressed only in PSWKBGD-3 in comparison to PSWKBGH-1 and 2 at 24*hai*, 48*hai* and 7*dai* (Supplementary Fig. 4). Figure 6 shows all the DEGs from each category taken from Supplementary Fig. 4, expressed in PSWKBGD-3 in comparison to PSWKBGH-1 and 2 combined.

**Fig. 7:**
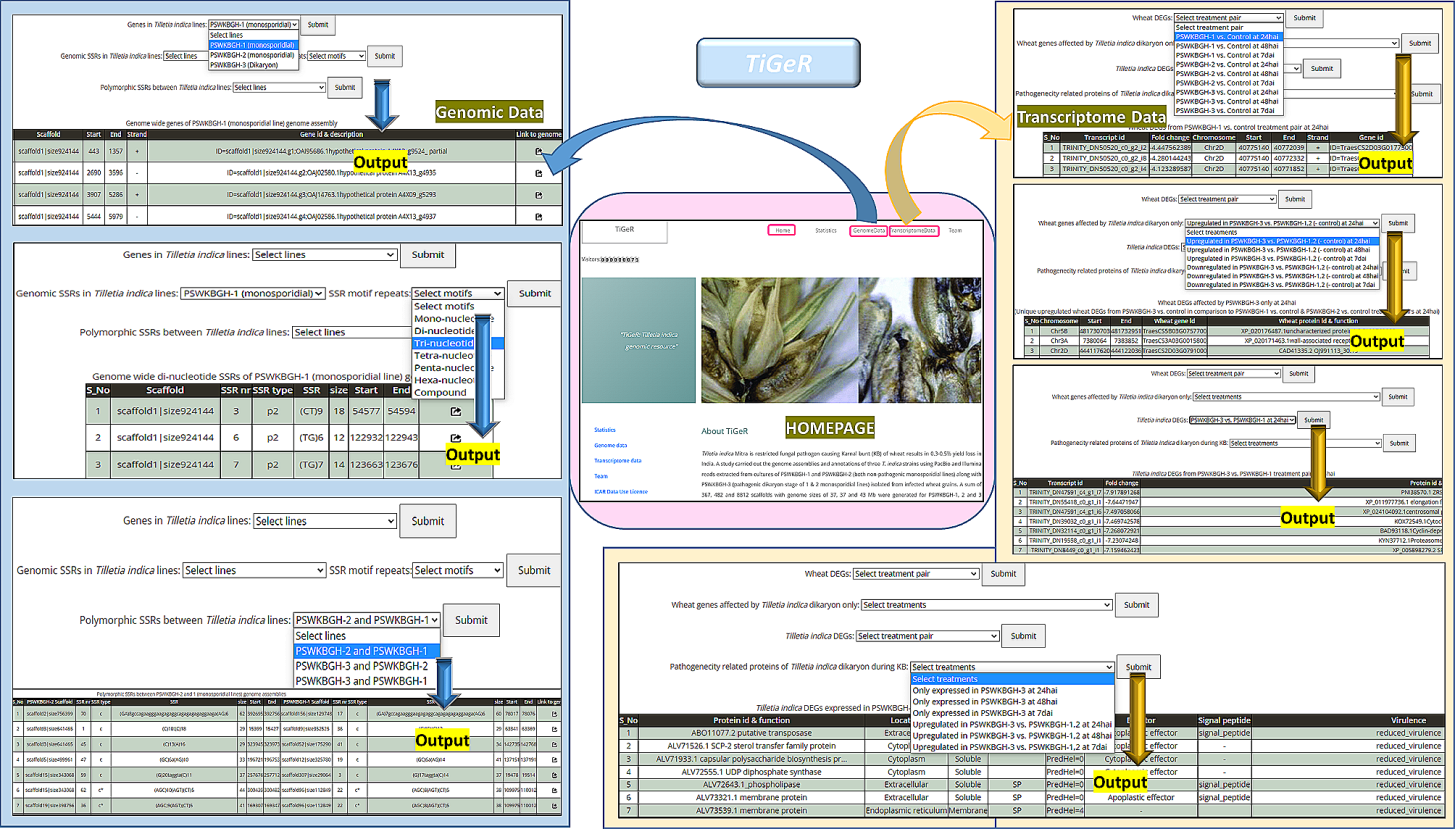
Web interface showing applications of *TiGeR* (*T.indica* genomic resource) for retrieving information

Functional annotation was also performed for *T. indica* up- and down-regulated DEGs, which were expressed only in dikaryon PSWKBGD-3 with respect to PSWKBGH-1 and 2 at 24*hai* (Fig. 6A), 48*hai* (Fig. 6B) and 7*dai* (Fig. 6C). Table 4 shows up- and down-regulated DEGs in PSWKBGD-3, along with DEGs only expressed in PSWKBGD-3 during transitions between different times.

**Table 4:** Important *T. indica* genes expressed only in PSWKBGD-3, up- and down-regulated in PSWKBGD-3 during progression of Karnal Bunt in wheat at different transitions of time (genes in bold are pathogenesis related genes of *T. indica* with their effects, detail of which is given in Supplementary Table 5)

Expression	Transitions	*T. indica* DEGs activated during Karnal bunt (pathogenesis related in bold with their effect)
Expressed only in PSWKBGD-3	24*hai*	Hypothetical protein (avirulence determinant)
	48*hai*	TIGR00156 family protein (hypervirulence)
	7*dai*	Leucine rich protein (avirulence determinant); S-phase-specific ribosomal protein (avirulence determinant); Type 2 metallothionein-like protein (hypervirulence); Hypothetical protein (avirulence determinant); 40s ribosomal protein (lethal)
	24*hai*, 48*hai* (04)	Pore-forming toxin-like protein Hfr-2; Factor of DNA methylation 5-like; Jasmonate-induced protein-like; Hypothetical protein
	48*hai*, 7*dai* (03)	Eukaryotic translation initiation factor 5 A-2; Two Uncharacterised proteins
	24*hai*, 7*dai* (06)	Probable Hmp1-Mismatch base pair and cruciform DNA recognition protein (lethal); TonB-dependent receptor; Argininosuccinate synthase; WYL domain-containing protein; Hypothetical protein; Uncharacterised protein;
Up-regulated in PSWKBGD-3	24*hai*	Ribulose bisphosphate carboxylase small chain C (lethal); Elongation factor P hydroxylase (hypervirulence); Uncharacterised protein (avirulence determinant)
	48*hai*	TonB family domain protein (hypervirulence); Septum formation protein Maf (hypervirulence); Sulfate adenylyltransferase (hypervirulence); Biopolymer transporter ExbB (avirulence determinant), General secretion pathway protein GspK (avirulence determinant); Zinc protease (hypervirulence); 5-formyltetrahydrofolate cyclo-ligase (lethal); Terminase (hypervirulence); Elongation factor P hydroxylase (hypervirulence); Protein/domain associated with GTPases (hypervirulence); 08 Uncharacterised proteins (01 lethal, 04 avirulence determinant, 03 hypervirulence), DcaP-like protein (hypervirulence); Signal peptide (avirulence determinant)
	24*hai*, 48*hai*, 7*dai* (10)	Alkyl hydroperoxide reductase subunit C; Lytic transglycosylase domain-containing protein; Iron-containing alcohol dehydrogenase; NADH-quinone oxidoreductase subunit NuoN; LysM peptidoglycan-binding domain-containing protein; Alkene reductase; Flavodoxin-dependent (E)-4-hydroxy-3-methylbut-2-enyl-diphosphate synthase; ATP-dependent metallopeptidase FtsH/Yme1/Tma family protein; Lipid A hydroxylase LpxO
	24*hai*, 7*dai* (10)	Protein CHROMATIN REMODELING 24 isoform X2; Dihydrolipoyl dehydrogenase; Sodium: proton antiporter; Retrotransposon protein; Ty1-copia subclass; Aspartokinase 1; Conserved protein; Uncharacterised protein; Tape measure protein; Cation transporter, Protein FAR1-RELATED SEQUENCE 5
	24*hai*, 48*hai* (99)	-
	48*hai*, 7*dai* (285)	50 S ribosomal protein L10 (hypervirulence), Nitrogen regulatory protein P-II (avirulence determinant); Uncharacterised protein (lethal)
Downregulated in PSWKBGD-3	24*hai*, 48*hai*, 7*dai* (02)	Protein TAR1; Uncharacterised protein
	24*hai*, 7*dai* (04)	Putative late blight resistance protein homolog R1B-16; Polyphosphate kinase 1; Proteasome subunit alpha type-4; Conserved protein
	48*hai*, 7*dai* (13)	Bbeta-lactamase TEM-1; NADH-cytochrome b5 reductase; Bowman-Bike type proteinase inhibitor A; Cell wall-associated hydrolase; Low quality protein; Three Conserved proteins; Three Hypothetical proteins; Three Uncharacterised proteins
	24*hai*, 48*hai* (11)	Chromatin remodeling 24 isoform X2; Putative disease resistance protein RGA3; Salt stress-induced protein; Zinc finger protein 454 isoform 2; Cyclin-dependent kinase variant; Disease resistance protein RPP13-like; Putative chitin-inducible gibberellin-responsive protein; Translation elongation factor eEF1, gamma chain; Powdery mildew resistance-related protein; Aspartokinase 1, chloroplastic-like isoform X1; Cathepsin B-like protease 3 isoform X3

### Putative pathogenicity related genes of dikaryon PSWKBGD-3 causing Karnal bunt

DEGs expressed only in PSWKBGD-3 compared to PSWKBGH-1 and 2 underwent various analysis to find their pathogenic nature, some of them are shown in bold Table 4. Supplementary Table 5 shows a sum of predicted effectors/secretory proteins; which could be putative pathogenesis related genes, up-regulated and expressed only in PSWKBGD-3. Supplementary Table 6 shows putative pathogenicity related genes in detail, that played a role in/as hypervirulence, plant avirulence determinant or lethal from PHI-base along with results from DeepLoc, SignalP, TargetP, TMHMM, and EffectorP.

### TiGeR: *T. indica* genomic resource

*T. indica* genomic resource (TiGeR) (Fig. 7 shows its layout) has five tabs namely Home, Statistics, Genome Data, Transcriptome Data, and Team. This is freely accessible at http://backlin.cabgrid.res.in/tiger/. The “Home” page provides a brief introduction to this web resource and provides tabs to navigate to various pages. The “Statistics” page contains the general detailed statistics of the PSWKBGH-1, 2, and PSWKBGD-3 assemblies. The “Genome Data” page navigates users to obtain the assembly-wise genes and genomic SSRs along with polymorphic SSRs among all three assemblies. The “Transcriptome Data” page provides detailed information on the wheat transcriptome DEGs, including its position on the chromosome, the fold change value and the gene ID for different sample combinations. Also, the wheat genes affected by *T. indica* dikaryon stage, the DEGs of *T. indica* at different time points can be retrieved from this tab. In addition, pathogenicity related proteins of *T. indica* dikaryon found during KB for different treatment combinations with detailed information of location, type of protein, effector and signal peptides as well as level of virulence.

## Discussion

The present study is the first of its kind including genome assembly of dikaryon (PSWKBGD-3) of *T. indica* and its monosporidial lines (PSWKBGH-1 and 2) along with two separate transcriptome analyses for wheat and *T. indica* transcripts during progression of KB infection in wheat by dikaryon (PSWKBGD-3) of *T. indica* in comparison to its monosporidial lines (PSWKBGH-1 and 2).

In the first part of the present study, *de novo* genome assembly of dikaryon (PSWKBGD-3) of *T. indica* and its monosporidial lines (PSWKBGH-1 and 2) was performed. Monosporidial lines of *T. indica* are non-pathogenic, although pathogenesis caused for the development of KB in wheat occurs only after the formation of dikaryon due to the conjugation of mycelia of two compatible monosporidial lines. However, all previous studies involving draft genome and analysis of pathogenesis of *T. indica* during KB in wheat were either based on monosporidial line(s) [10, 15, 17–19, 47, 48], and/or on hyphae germinated from teliospores. Previously, the genome assemblies of PSWKBGH-1 and 2 were announced in 2016 [13]. However, in the present study, along with genome assembly of dikaryon (PSWKBGD-3) causing pathogenesis during KB in wheat and its two compatible monosporidial lines (PSWKBGH-1 and 2), a comprehensive analysis of the assembled genomes was also performed. Assembled genome of PSWKBGD-3 showed better quality assessment and larger genome in comparison to genomes of PSWKBGH-1 and 2. This confirms the existence of PSWKBGD-3 as the dikaryon of PSWKBGH-1 and 2. Further, the SSR mining report from three genome assemblies showed a sufficient number of SSRs to be monomorphic and polymorphic along with unique SSRs to each assembly. These polymorphic and unique SSRs can be used for marker-trait association and diversity analysis in genetic and population studies. Unique SSRs of the PSWKBGD-3 genome assembly can be used as markers for the detection of KB in wheat, since it is difficult to detect KB in the early development stage of wheat. Gurjar et al., 2022a [17] reported 5772 SSRs, which is comparable to the number of SSRs extracted from genome assemblies of PSWKBGH-1, 2 and PSWKBGD-3 in the present study. Similarly, the frequency of tri-nucleotide SSRs, followed by mono and di-nucleotide repeat SSRs in PSWKBGH-1, 2 and PSWKBGD-3 in the present study is in corroboration with the previous study [17]. This is the first study providing genome-wide polymorphic SSRs of *T. indica* among three lines, however, Gurjar et al., 2022a [17] reported 18 highly informative polymorphic SSRs of *T. indica* out of 40 SSRs used. Later, genome annotation was also performed in the present study and submitted, which was absent in previous studies on *T. indica* [13, 47].

In the second part of the present study, two transcriptome analyses were performed utilizing *T. indica* transcriptome from PSWKBGH-1, 2 and PSWKBGD3 to extract dikaryon (PSWKBGD-3) genes responsible for pathogenesis, and wheat transcriptome to extract wheat genes affected by dikaryon (PSWKBGD-3) involved in plant-pathogen interaction during progression of KB in wheat. However, a few previous studies were found involving KB resistance [50–53] or identification of *T. indica* pathogenesis related genes [16, 52] in wheat using genomic data. Gurjar et al., 2018b [11] studied regulation of genes from *T. indica* grown from germination of teliospores, means utilized monosporidial hyphae to extract RNA. Recently, Gurjar et al., 2022b [19] provided a transcriptome analysis of RAKB_UP_1 isolate of *T. indica* utilizing draft genome of the same isolate. However, the present study utilized pure dikaryon hyphae (PSWKBGD-3) and hyphae of its monosporidial lines (PSWKBGH-1, 2) to inoculate seeds of a susceptible wheat cultivar (WL711), then, RNA was isolated from the wheat seeds inoculated by hyphae of dikaryon and its monosporidial lines to perform two transcriptome analyses for both wheat and *T. indica* transcripts to get the exact effect of dikaryon on wheat gene expression and genes of dikaryon important for KB progression. In the present study, the transcriptome analysis of wheat during KB, reports that more wheat genes are affected due to pathogenesis caused by PSWKBGD-3 in comparison to monosporidial lines PSWKBGH-1 and 2, and were down-regulated at 24*hai* and 48*hai*, and upregulated at 7*dai* during the progression of KB.

During plant-pathogen interaction, the pathogen has to pass through two defense layers in plants. The first layer of defense is at the plasma membrane level, where pathogen-associated molecular patterns (PAMPs) or apoplastic effectors of the pathogen are sensed by host plasma membrane receptors. To initiate pathogenesis, PAMPs or apoplastic effectors must overcome PAMP-triggered immunity (PTI) triggered by subsequent intracellular signal transduction events. The second layer of defense is at the cellular level, where intracellular avirulence (Avr) effectors are recognized by proteins encoded by host resistance genes (R-genes), which induce effector-triggered immunity (ETI) [54, 55]. In the present study, it was found that most of the wheat genes responsible for KB resistance in wheat causing PTI and ETI were down-regulated during the growth of dikaryon (PSWKBGD-3), which is responsible for the development of the disease. Some of the previously reported KB resistant genes were found to be downregulated in transcriptome of wheat during KB infection and its progression in the present study as the wheat cultivar, WL711, utilized in the present study is susceptible to KB. These genes were ABC transporter, NAC domain-containing protein, Disease resistance proteins, Glutathione S-transferase, Wall-associated receptor kinase, Protein kinase. Some of the other down-regulated wheat genes were protein DETOXIFICATION 16 and NBS-LRR protein and up-regulated were ATP synthase subunit C family protein, 6-phosphofructokinase 2, Glycosyltransferase, HGA-like, xyloglucan endotransglycosylase/hydrolase protein 8 etc.

It was also found that more PSWKBGD-3 genes were up-regulated in comparison to PSWKBGH-1 and 2 during 24*hai*, 48*hai* and 7*dai*. Higher number of genes was up-regulated during 48*hai*, which suggests that after crossing the PTI post establishment of the plant-pathogen interaction, a large number of genes were needed to upregulate to suppress the ETI at 48*hai* for the disease progression. However, there was a continuous increase in activation of PSWKBGD-3 genes, expressed only in PSWKBGD-3 dikaryon stage from 24*hai* to 7*dai* and more unique sets of genes were activated at each time stage. Few genes activated only in PSWKBGD-3, may have been involved in the transition from one time phase to another during the progression of KB, e.g., Pore-forming toxin-like protein Hfr-2, Factor of DNA methylation 5-like, Jasmonate-induced protein-like during transition from 24*hai* to 48*hai*; Eukaryotic translation initiation factor 5 A-2 from 48*hai* to 7*dai*; Hmp1-Mismatch base pair and cruciform DNA recognition protein, TonB-dependent receptor, Argininosuccinate synthase, and WYL domain-containing protein from 24*hai* to 7*dai*.

Further, *T. indica* genes only activated in PSWKBGD-3 and up-regulated in PSWKBGD-3 compared to PSWKBGH-1 and 2 monosporidial lines were analysed to find pathogenicity related genes having role as effector plant avirulence determinant, hypervirulence and lethal genes; which could be putative PAMPs and Avr effectors activated and upregulated during plant-pathogen interaction in progression of KB. Effectors could act in the early infection to suppress PTI and achieve initial establishment and/or rewire host defence signaling and cellular activities for pathogen proliferation and nourishment within the host cells [54, 55]. Some of the putative pathogenicity related genes of PSWKBGD-3 extracted from this study, were activated during different time phases and further their role was also accessed to check that whether they are suppressing the PTI as avirulence determinant to surpass the first level of host defense to establish the KB or ETI as hypervirulence effector to surpass second level of host defense in progression of KB or lethal to ultimately causing the death of cell and further death of the host due to KB. Such extracted genes were Ribulose bisphosphate carboxylase small chain C- chloroplast precursor, Elongation factor P hydroxylase at 24*hai*; TIGR00156 family protein, Signal peptide, TonB family domain protein, DcaP-like protein, Septum formation protein Maf, Sulfate adenylyl transferase, small subunit, Biopolymer transporter ExbB, General secretion pathway protein GspK, Zinc protease, 5-formyltetrahydrofolate cyclo-ligase, Terminase, Elongation factor P hydroxylase, Protein/domain associated with GTPases at 48*hai*; Leucine rich protein, S-phase-specific ribosomal protein, Type 2 metallothionein-like protein, 40s ribosomal protein at 7*dai* along with Hmp1-Mismatch base pair and cruciform DNA recognition protein, 50 S ribosomal protein L10, and Nitrogen regulatory protein P-II during 48*hai* and *7dai*. It was also found that some hypothetical and uncharacterized proteins were also play a very significant role in pathogenesis during incidence of disease.

The findings of this study would be helpful in understanding the plant-pathogen interaction during progression of pathogenesis of KB in wheat involving the dikaryon stage of *T. indica*. A web-resource, *TiGeR* for *T. indica*, was developed to include results from this study, including genomic data of the dikaryon (PSWKBGD-3) and its monosporidial lines (PSWKBGH-1 and 2) along with transcriptome data of both *T. indica* and wheat during progression of KB. *TiGeR* is the first *T. indica* genomic resource that could be utilized by the research community for the management of *T. indica* in wheat and development of KB resistant wheat varieties.

## Conclusion

The present study provides the first draft genome assembly of the pathogenic dikaryon (PSWKBGD-3) of *T. indica* causing KB in wheat, with 8812 scaffolds with a size of 43 Mb compared to its monopodial lineages (PSWKBGH-1 and 2) containing 367 and 482 scaffolds of 37 Mb each along with genome scoring and annotation that could be used in genomic studies of pathogenesis during KB. The present study also reports for the first time 817 genome-wide polymorphic SSRs in all three genome assemblies of PSWKBGH-1, 2, and PSWKBGD-3, which could be used in genetic diversity and lineage specification studies. In the second part of the present study, two transcriptome analyses were performed utilizing *T. indica* transcriptome from PSWKBGH-1, 2 and PSWKBGD3 to extract dikaryon (PSWKBGD-3) genes responsible for pathogenesis, and wheat transcriptome to extract wheat genes affected by dikaryon (PSWKBGD-3) involved in plant-pathogen interaction during progression of KB in wheat. Few, 08 putative pathogenesis-related genes (act as hypervirulence/avirulence determinant/lethal genes) were extracted that were activated only in dikaryon PSWKBGD-3 during KB progression, and other 26 such genes were upregulated in the dikaryon PSWKBGD-3 compared to its monosporidial lineages during KB progression at 24*hai*, 48*hai* and 7*dai*. The present study also reports 85 wheat genes affected by dikaryon (PSWKBGD-3) during KB infection in wheat extracted from wheat transcriptome. This study contributes significantly to the understanding of *T. indica* as well as wheat genes affected by the pathogenic dikaryon stage involved in host-pathogen interaction during KB. This will help better management of KB in wheat, pave the way for the development of KB resistant wheat varieties, and ensure food security around the world.

### Electronic supplementary material

Below is the link to the electronic supplementary material.

Supplementary Material 1

## Data Availability

The three genome assemblies are available on NCBI as project id PRJNA325874, i.e., PSWKBGH-1 (GCA_001689995.1), PSWKBGH-2 (GCA_001689945.1) and PSWKBGD-3 (GCA_001689965.1 (https://www.ncbi.nlm.nih.gov/bioproject/PRJNA325874). Transcriptome data is available under project id: PRJNA878612 along with all library IDs: SAMN30733936-SAMN30733947.
