# Supplementary material for: Genome and transcriptome based comparative analysis of Tilletia indica to decipher the causal genes for pathogenicity of Karnal bunt in wheat
Source: BMC Plant Biol. 2024 Jul 16;24:676. doi: 10.1186/s12870-024-04959-z (PMC11251232; doi:10.1186/s12870-024-04959-z)

**Supplementary materials**

Supplementary Table 1: Details of Illumina and PacBio libraries of monosporidial lines and dikaryon stage of *T. indica*

| Libraries in single replicate | Illumina – NextSeq (150*2) reads | PacBio reads |
| --- | --- | --- |
| PSWKBGH-1 (Monosporidial) | 10913793 | 170346 |
| PSWKBGH-2 (Monosporidial) | 10106154 | 227135 |
| PSWKBGD-3 (Dikaryon) | 21463265 | - |

Supplementary Table 2: Summary of SSRs in monosporidial lines (PSWKBGH-1 and 2) of *T. indica* and their dikaryon (PSWKBGD-3)

| Assembly | SSRs | Mono | Di | Tri | Tetra | Penta | Hexa | Compound |
| --- | --- | --- | --- | --- | --- | --- | --- | --- |
| PSWKBGH-1 | 6347 | 1794 | 1428 | 2404 | 189 | 43 | 121 | 368 |
| PSWKBGH-2 | 6164 | 1814 | 1366 | 2217 | 166 | 53 | 147 | 401 |
| PSWKBGD-3 | 6938 | 1625 | 1649 | 2641 | 223 | 56 | 136 | 608 |

Supplementary Table 3: Detail of RNA-seq libraries of affected susceptible wheat cultivar WL711 inoculated with monosporidial lines (PSWKBGH-1 and 2) and dikaryon stage (PSWKBGH-3) of *T. indica* along with Control at 24*hai*, 48*hai* and 7*dai* in replicates (R1 and R2)

| RNA-seq Libraries | Raw reads | Cleaned reads | GC% | Transcripts |
| --- | --- | --- | --- | --- |
| PSWKBGH-1:24*hai** (R1, R2) | 95638446, 85964520 | 6324564, 4873448 | 47.5, 48 | 136230 |
| PSWKBGH-1:48*hai* (R1, R2) | 80842810, 97391514 | 4698208, 2239274 | 48, 48 | 106568 |
| PSWKBGD-1:7*dai* (R1, R2) | 96741264, 101944430 | 2071064, 2734088 | 53, 44 | 72296 |
| PSWKBGH-2:24*hai* (R1) | 74869602 | 2466482 | 48 | 50118 |
| PSWKBGH-2:48*hai* (R1, R2) | 71820528, 79358902 | 3171830, 2311828 | 48, 46 | 103949 |
| PSWKBGD-2:7*dai* (R1, R2) | 91149730, 90982820 | 1897508, 2348800 | 49, 49 | 96295 |
| PSWKBGH-3:24*hai* (R1, R2) | 84526578, 98120162 | 3720808, 3624440 | 48, 49 | 113013 |
| PSWKBGH-3:48*hai* (R1, R2) | 77756640, 73218920 | 4116118, 3728922 | 47, 48 | 111265 |
| PSWKBGD-3:7*dai* (R1, R2) | 97576946, 96839512 | 2612610, 2473006 | 44.5, 44 | 92897 |
| Control:24*hai* (R1) | 118802530 | 1782540 | 50 | 44018 |
| Control:48*hai* (R1) | 121541016 | 2722350 | 50 | 49445 |
| Control:7*dai* (R1) | 127119696 | 3455332 | 43.5 | 49641 |

**hai* and *dai*= hours and days after inoculation; R1 and R2=replicate 1 and 2

Supplementary Table 4: The upregulated and downregulated DERs and DEGs of susceptible wheat cultivar WL711 inoculated with monosporidial lines (PSWKBGH-1 and 2) of *T. indica* and their dikaryon (PSWKBGD-3) in comparison to control at 24*hai*, 48*hai* and 7*dai*

| Treatment pairs | DERs | | | DEGs | | |
| --- | --- | --- | --- | --- | --- | --- |
|  | All | Up* | Down | All | Up | Down |
| PSWKBGH-1 vs. Control:24*hai* | 9260 | 5936 | 3324 | 158 | 84 | 74 |
| PSWKBGH-1 vs. Control:48*hai* | 10809 | 7604 | 3205 | 237 | 168 | 69 |
| PSWKBGH-1 vs. Control:7*dai* | 6888 | 1305 | 5583 | 113 | 26 | 87 |
| PSWKBGH-2 vs. Control:24*hai* | 17575 | 9726 | 7849 | 290 | 172 | 118 |
| PSWKBGH-2 vs. Control:48*hai* | 13187 | 8740 | 4447 | 260 | 183 | 77 |
| PSWKBGH-2 vs. Control:7*dai* | 15945 | 13090 | 2855 | 313 | 223 | 90 |
| PSWKBGD-3 vs. Control:24*hai* | 10363 | 5571 | 4792 | 173 | 85 | 88 |
| PSWKBGD-3 vs. Control:48*hai* | 7483 | 4903 | 2580 | 114 | 69 | 45 |
| PSWKBGD-3 vs. Control:7*dai* | 7773 | 5975 | 1798 | 140 | 104 | 36 |

### *Upregulated and downregulated DERs/DEGs

Supplementary Table 5: Putative pathogenicity related genes as secretory proteins and effectors, expressed only and upregulated in PSWKBGD-3 during progression of pathogenesis by *T. indica* in wheat at different time durations

| Expression | Time | Predicted effectors/secretory proteins |
| --- | --- | --- |
| Expressed only in PSWKBGD-3 | 24*hai* | 21 |
|  | 48*hai* | 35 |
|  | 7*dai* | 134 |
| Upregulated in PSWKBGD-3 | 24*hai* | 57 |
|  | 48*hai* | 529 |
|  | 7*dai* | 87 |

Supplementary Table 6: Putative pathogenicity related genes from PHI-base expressed only and upregulated in PSWKBGD-3 during progression of pathogenesis by *T. indica* in wheat at different time durations

| Expression | Time | Effectors with hypervirulence/ plant avirulence determinant/ lethal (cellular location, Helix, effector/signal peptide, virulence) |
| --- | --- | --- |
| Expressed only in PSWKBGD-3 | 24*hai* | ENV71128.1 hypothetical protein (cTP*, Hel**=0, effector plant avirulence determinant) |
|  | 48*hai* | EEY90864.1 putative TIGR00156 family protein (SP***, Hel=0, Cytoplasmic effector, hypervirulence) |
|  | 7*dai* | EFD66348.1 Leucine rich protein (cTP, Hel=0, Apoplastic effector, effector plant avirulence determinant), AAC98779.1 S-phase-specific ribosomal protein (Nucleus, Hel=0, Cytoplasmic effector, effector plant avirulence determinant), AXP11719.1 type 2 metallothionein-like protein (cytoplasm, Hel=0, Apoplastic/cytoplasmic effector, hypervirulence), KFA45286.1 hypothetical protein (Extracellular, Hel=0, Apoplastic effector, plant avirulence determinant), KIY50194.1 40s ribosomal protein (Cytoplasm, Hel=0, Cytoplasmic effector, lethal) |
|  | 48*hai*, 7*dai* | CDU24116.1 Probable Hmp1-Mismatch base pair and cruciform DNA recognition protein (Cytoplasm, Hel=0, Cytoplasmic effector, lethal) |
| Upregulated in PSWKBGD-3 | 24*hai* | ABG21958.1 Ribulose bisphosphate carboxylase small chain C, chloroplast precursor (cTP, Hel=0, lethal), SJX22175.1 Elongation factor P hydroxylase (cytoplasm, Hel=0, Cytoplasmic effector, Hypervirulence), SPW29625.1 Uncharacterised protein (Nucleus, Hel=0, Cytoplasmic effector, effector plant avirulence determinant) |
|  | 48*hai* | EEY95365.1 TonB family domain protein (Golgi apparatus , Hel=0 Cytoplasmic effector, Hypervirulence), EEY95822.1 septum formation protein Maf (Cytoplasm, Hel=0, Cytoplasmic effector, Hypervirulence), EEY97490.1 sulfate adenylyltransferase, small subunit (Cytoplasm, Hel=0, Cytoplasmic effector, Hypervirulence), OFW90794.1 biopolymer transporter ExbB (SP, Hel=0, effector plant avirulence determinant), OHC23801.1 general secretion pathway protein GspK (SP, Hel=1, effector plant avirulence determinant), PPE76582.1 zinc protease (Mitochondrion, Hel=0, Cytoplasmic effector, Hypervirulence), PZO93138.1 5-formyltetrahydrofolate cyclo-ligase (Nucleus, Hel=0, Cytoplasmic effector, lethal), RZN87725.1 terminase (Cytoplasm, Hel=0, Cytoplasmic effector, Hypervirulence), SJX22175.1 Elongation factor P hydroxylase (Cytoplasm, Hel=0, Cytoplasmic effector, hypervirulence), SNU13456.1 putative protein/domain associated with GTPases (Peroxisome, Hel=0, Cytoplasmic effector, Hypervirulence), SNU13707.1 Uncharacterised protein (SP, Hel=0, Cytoplasmic effector, lethal), SNU15599.1 Uncharacterised protein (SP, Hel=0, Cytoplasmic effector, effector plant avirulence determinant), SNU15921.1 Uncharacterised protein (SP, Hel=0, Cytoplasmic/apoplastic effector, hypervirulence), SNU16100.1 Uncharacterised protein (Cytoplasm, Hel=0, Cytoplasmic effector, effector plant avirulence determinant), SUT92587.1 Uncharacterised protein (Cytoplasm, Hel=0, Cytoplasmic effector, effector plant avirulence determinant), SUT93101.1_putative_DcaP-like_protein (SP, Hel=0, hypervirulence), SUT96490.1 Uncharacterised protein (Cytoplasm, Hel=0, Cytoplasmic effector, hypervirulence), SUT98246.1 Uncharacterised protein (SP, Hel=2, effector plant avirulence_determinant), SUT99945.1 Signal peptide(SP, Hel=1, effector plant avirulence determinant) |
|  | 48*hai*, 7*dai* | CAG67265.1 50S ribosomal protein L10 (Cytoplasm, Hel=0, Cytoplasmic effector, hypervirulence), EEH69901.1 nitrogen regulatory protein P-II (Cytoplasm, Hel=0, Cytoplasmic effector, effector, plant avirulence determinant), SNU14480.1 Uncharacterised protein (SP, Hel=0, Cytoplasmic effector, lethal) |

*cTP= chloroplast transit peptide; **Hel=Helix; ***SP=Signal peptide

**Supplementary Figures**

Supplementary Figure 1: Confocal microscope images showing (a) *T. indica* hyphae in wheat leaf, (b) Electron microscope image of fungal hypae and (c) spores


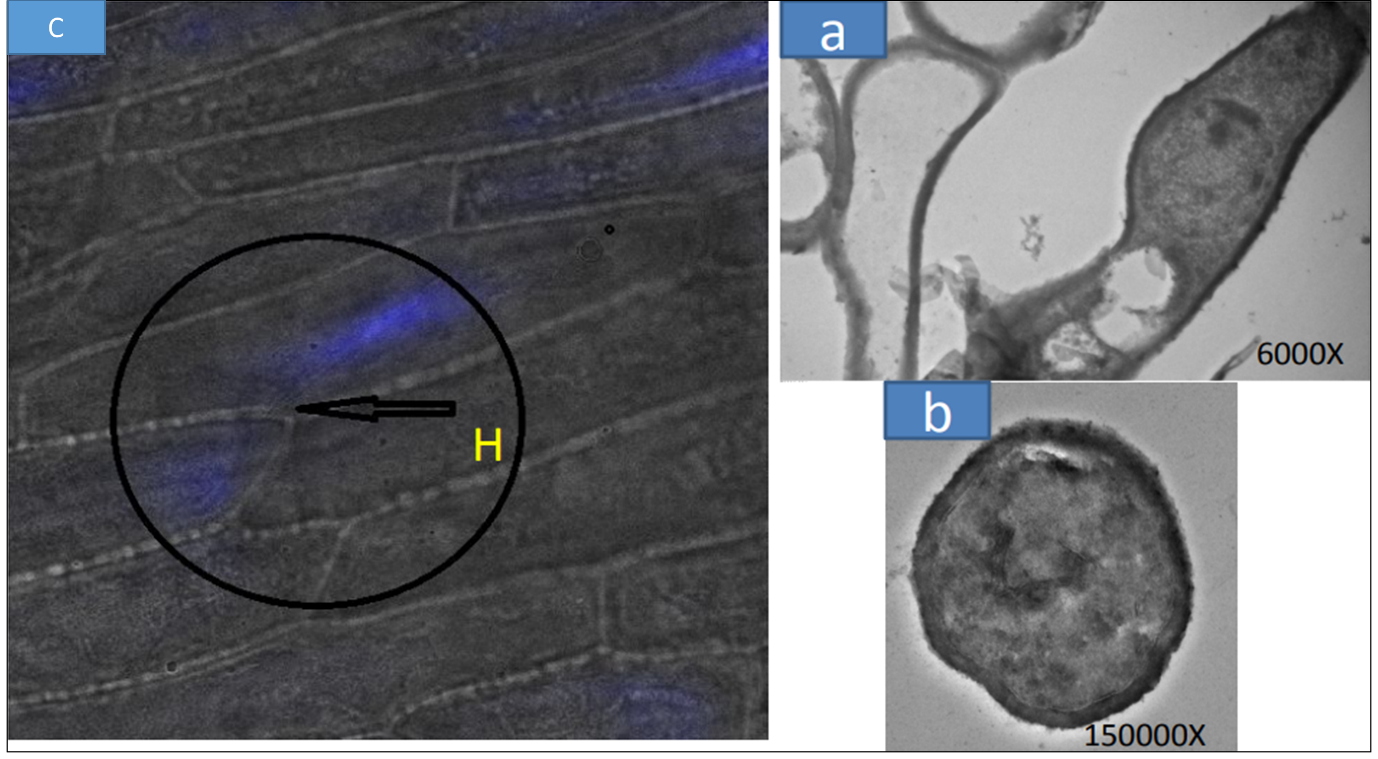


Supplementary Figure 2: All, upregulated and downregulated DERs of PSWKBGD-3 dikaryon of *T. indica* in comparison to PSWKBGH-1 and 2 monosporidial lines at 24*hai*, 48*hai* and 7*dai*


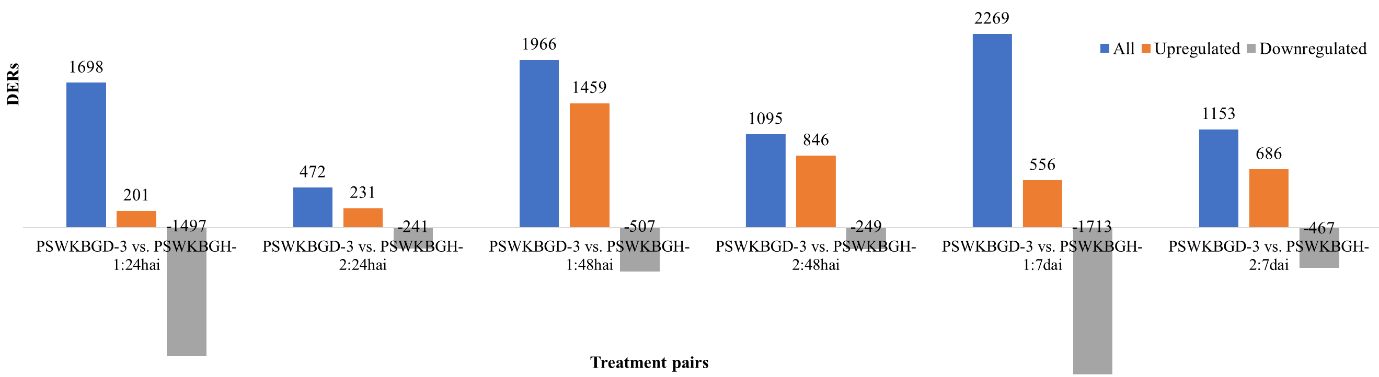


Supplementary Figure 3: All, upregulated and downregulated DEGs of PSWKBGD-3 dikaryon of *T. indica* in comparison to PSWKBGH-1 and 2 monosporidial lines at 24*hai*, 48*hai* and 7*dai*


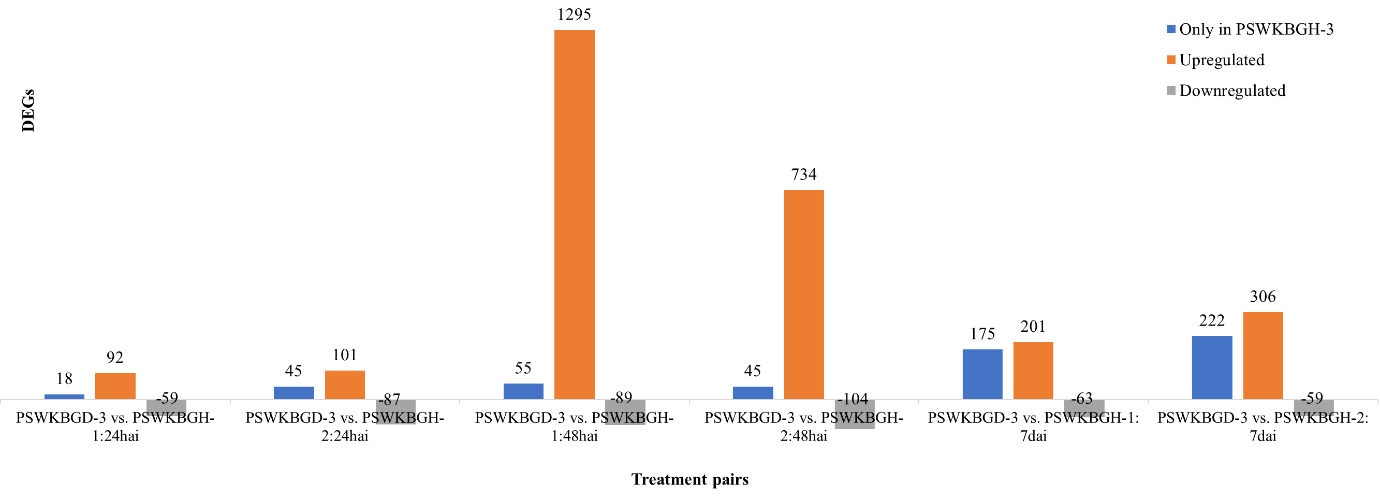


Supplementary Figure 4: Common *T. indica* DEGs in various treatment pairs (a) DEGs expressed only in PSWKBGD-3 dikaryon; (b) upregulated in PSWKBGD-3 dikaryon; (c) downregulated in PSWKBGD-3 dikaryon of *T. indica* in comparison to PSWKBGH-1 and 2 monosporidial lines at 24*hai*, 48*hai* and 7*dai*


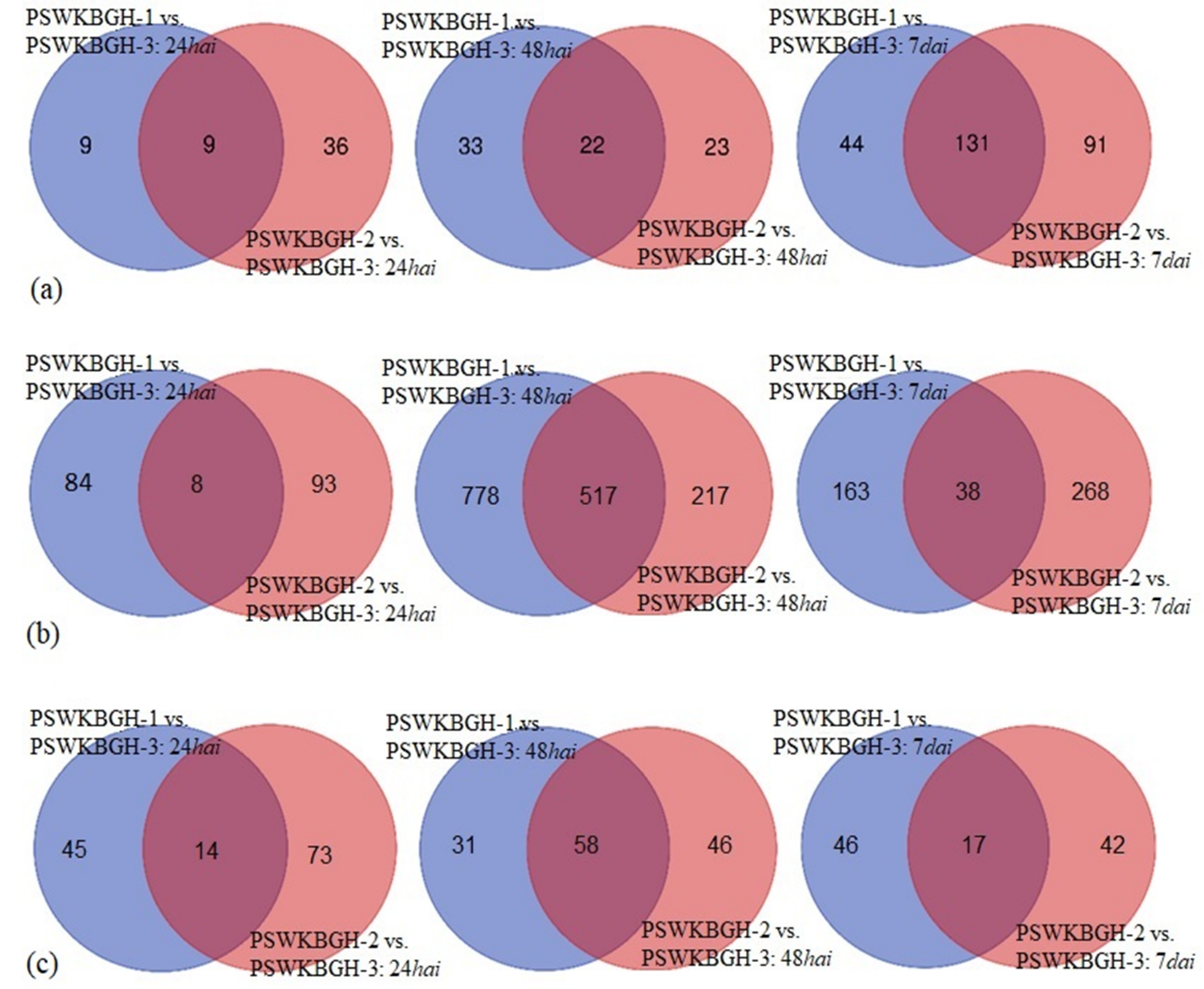

Supplement: Supplementary file 1 — Supplementary Material 1 [file 12870_2024_4959_MOESM1_ESM.docx]
